# Case Report: Nuchal Bursitis Associated With *Borrelia burgdorferi* Infection in a Horse

**DOI:** 10.3389/fvets.2021.743067

**Published:** 2021-09-23

**Authors:** Cassandra Guarino, Toby Pinn-Woodcock, David G. Levine, Julia Miller, Amy L. Johnson

**Affiliations:** ^1^Population Medicine and Diagnostic Sciences, Cornell University, Ithaca, NY, United States; ^2^New Bolton Center, University of Pennsylvania School of Veterinary Medicine, Kennett Square, PA, United States; ^3^General Medicine, Alliance Equine Health Care, Glenmoore, PA, United States

**Keywords:** lyme disease, poll evil, ospA, lyme multiplex assay, equine, ospF, antibody

## Abstract

Cases of cranial nuchal bursitis associated with *Borrelia burgdorferi* infection have not been thoroughly described. Here, we describe the case of a 17-year-old mare that was presented for low head carriage, dull demeanor, and resistance to haltering. Imaging supported a diagnosis of nuchal bursitis, and bursoscopy with surgical debridement of the nuchal bursa was performed. *B. burgdorferi* was identified by molecular diagnostics in serial samples of the bursal fluid, with no other organisms identified. Serology revealed significant elevation in antibodies directed against OspA of *B. burgdorferi*, but not the typical infection markers, OspC and OspF. Intravenous ceftiofur was administered for 80 days, and the nuchal bursa was directly injected with ceftiofur. The mare recovered and was able to return to work with no recrudescence of clinical signs over the following year to date. Infection with *B. burgdorferi* should be considered as a differential in cases of septic nuchal bursitis.

## Introduction

Cranial nuchal bursitis, colloquially known as “poll evil”, was originally thought to arise from trauma, but suspicion of a septic process was raised over a century ago (1). A variety of infectious causes have been implicated, including *Brucella abortus* (2), and both gram-negative and gram-positive bacteria have been cultured from bursal fluid samples from affected animals (3). Nuchal bursitis typically presents with head and neck pain and abnormal head carriage, sometimes with swelling over the poll and an associated draining tract (4). A retrospective study of 30 horses treated after a diagnosis of cranial nuchal bursitis revealed that samples from two horses tested positive by PCR for *B. burgdorferi*, but the significance of these findings was unclear (3).

*B. burgdorferi* sensu lato infection and Lyme disease in horses is a controversial topic in equine clinical practice due to the paucity of available evidence for disease pathologies, combined with the high seroprevalence for *B. burgdorferi* in horses residing in Lyme endemic regions (5). *B. burgdorferi* infection occurs through the bite of an infected tick and can subsequently spread through the bloodstream to a variety of tissues within the mammalian host (6). In horses, *B. burgdorferi* infection is commonly diagnosed with the Lyme multiplex assay (7), where positive antibody response to the outer surface protein (Osp) OspF is evidence of historic or chronic infection with *B. burgdorferi*. OspA antibodies are typically considered evidence of vaccination (8), but low transient OspA antibody values can be detected early in the course of equine infection, as is also reported in dogs (9, 10). Some horses will produce a robust and persistent OspA immune response after infection with *B. burgdorferi* (5, 11), but little is known about the cause or significance of this variation in immune response to *B. burgdorferi* in the horse.

OspA antibodies have been detected late in the course of human *B. burgdorferi* infection, but only in a minority of cases (12, 13). Further, in humans, development of OspA antibodies after infection with *B. burgdorferi* has been associated with the clinical presentation of arthritis, where higher IgG responses to OspA correlate with more severe and prolonged arthritis (14). Here, we present a case of equine nuchal bursitis where *B. burgdorferi* was identified by PCR in serial bursal fluid samples, and only OspA antibodies were produced in response to this infection.

## Case History

A 17-year-old, 600-kg Warmblood mare was presented to her primary care veterinarian with low head carriage, dull demeanor, and resistance to haltering after having been on cetirizine hydrochloride (All Day Allergy, GoodSense, New Brunswick, NJ, 0.2–0.3 mg/kg PO q12h) due to multiple episodes of full body hives over the preceding 2 weeks. Prior to the development of clinical signs, the mare had been routinely maintained on a supplement for allergy (Platinum Skin and Allergy, Platinum Performance, Buellton, CA, 5.3 mg/kg PO q12h) to control *Culicoides* hypersensitivity that the mare commonly experienced during spring–fall, altrenogest (Regu-Mate; Merck Animal Health, Kenilworth, NJ, 0.044 mg/kg PO q24h) for behavioral modification, initiated approximately 5 years prior, and pergolide (Prascend; Boehringer Ingelheim, Duluth, GA, 0.0017 mg/kg PO q24h) for management of pituitary pars intermedia dysfunction (PPID) diagnosed the preceding year. Physical examination was unremarkable except for a raised line that appeared to be a superficial vessel running from the nuchal bursa area caudally down the right side of the neck toward the vertebral column; this finding had been evident for several days. Initial hematology revealed lymphopenia (989 cells/μl; reference interval (RI) 1,500–5,500 cells/μl). Lyme multiplex assay was negative for antibodies against OspC and OspF but was markedly positive for antibodies against OspA (24,204 median fluorescent intensity (MFI); RI <2,000 MFI). Serum amyloid A (SAA) (StableLab Equine Blood Analysis Kit, Sligo, Ireland) was elevated based on qualitative colorimetric read-out. Treatment was initiated with flunixin meglumine (Banamine; Merck Animal Health, Madison, NJ, 1.1 mg/kg IV, once) and minocycline (Aurobindo, East Windsor, NJ, 4 mg/kg PO q12h). Cetirizine treatment was discontinued.

**Figure 1:**
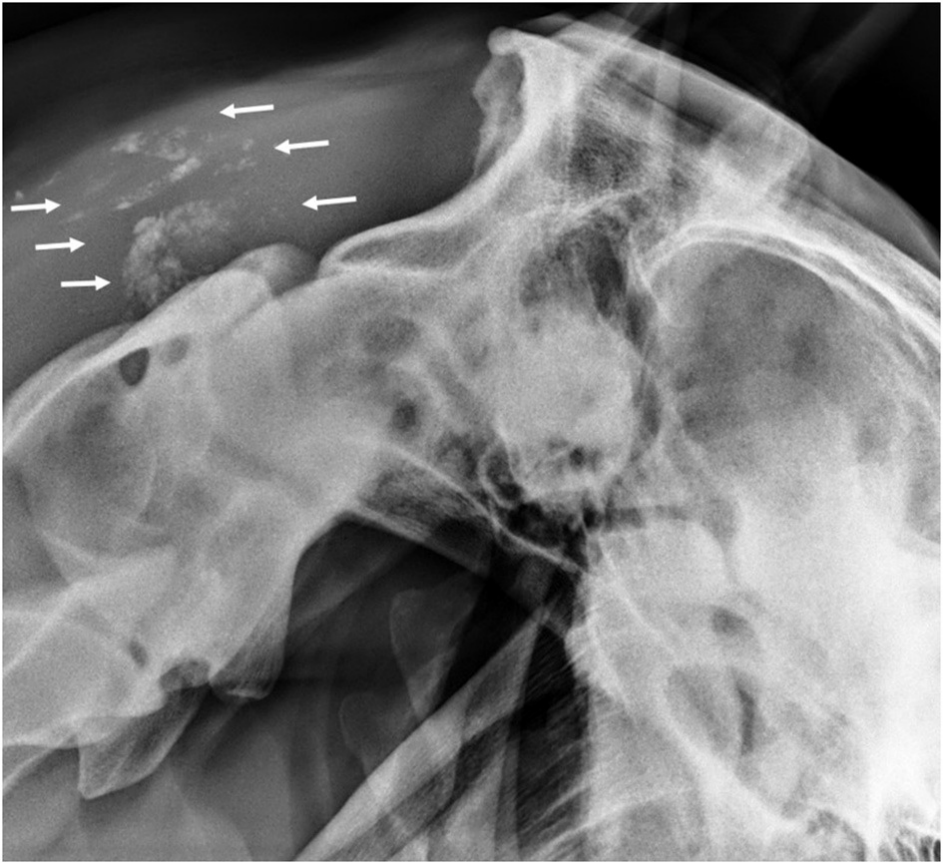
Lateral radiograph of the atlanto-occipital joint showing mineralization in the region of the nuchal bursa (arrows).

**Figure 2:**
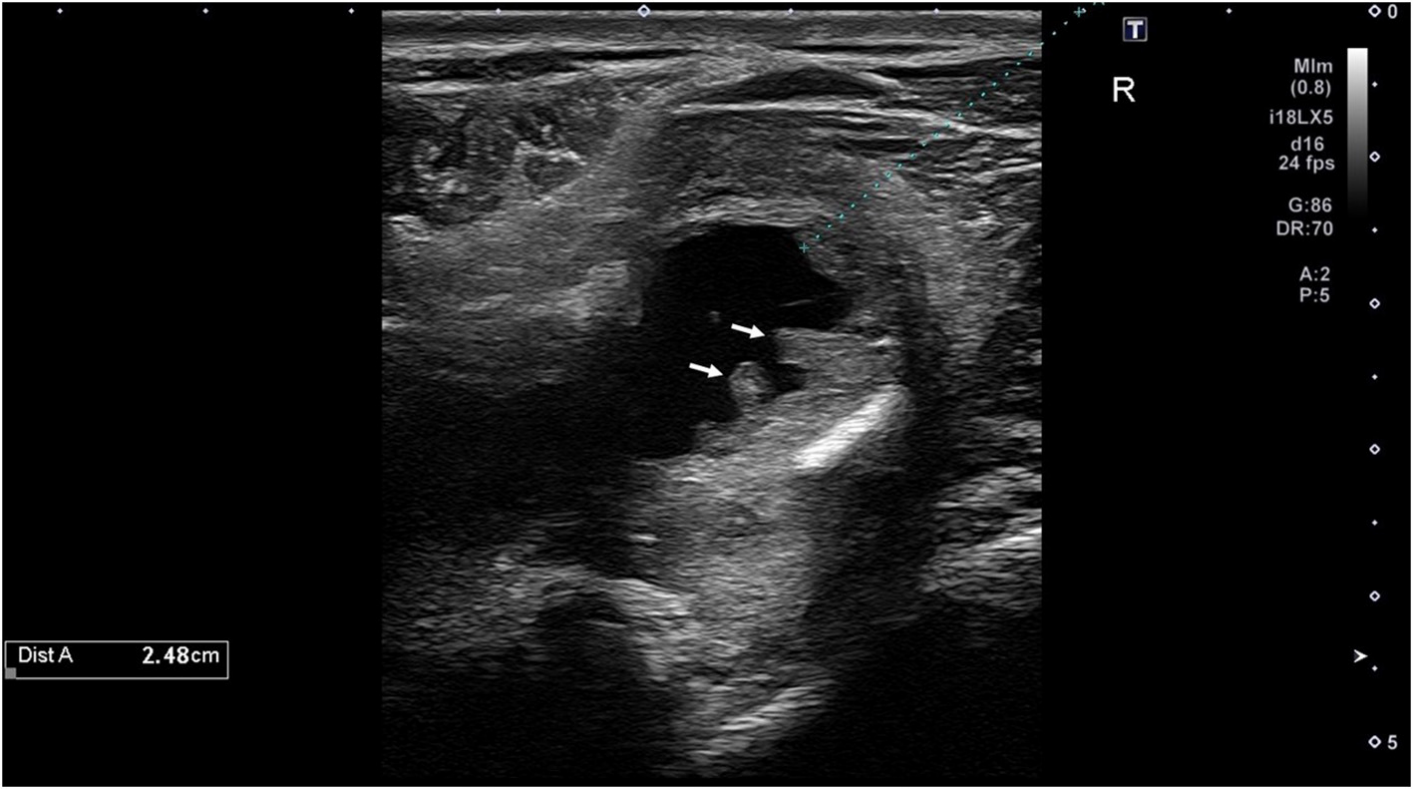
Ultrasound image of the cranial nuchal bursa showing marked distension with anechoic fluid and synovial proliferation (arrows).

Hives returned the following day and a single dose of dexamethasone (VetOne, Boise, Idaho, 0.03 mg/kg IV once) was administered, and hydroxyzine (Epic Pharma, Springfield Gardens, NY, 1.1 mg/kg PO, q12h) treatment was initiated. Although the mare initially improved clinically with decreased resentment to dorsoflexion of the neck under saddle, her head carriage continued to be abnormally low. Repeat hematology showed continued lymphopenia (1,071 cells/μl; RI 1,500–5,500 cells/μl) and the development of mild hyperfibrinogenemia (308 mg/dl; RI 76–230 mg/dl). Nine days after initiating treatment, SAA was negative, but the mare's abnormally low neck posture returned and the she exhibited pain on palpation of the poll. Minocycline was continued, the hydroxyzine dose was reduced (1 mg/kg, PO, q12h), and phenylbutazone (Vetribute, VetOne, Boise, Idaho, 2.2 mg/kg PO q12h) was administered. Radiographs (Figure 1) and ultrasound (Figure 2) of the poll area revealed ossification and mineralization dorsal to the first (C1) and second (C2) cervical vertebrae, as well as hyperechoic fluid in the cranial aspect of the nuchal bursa. Clinical signs progressed over the following week and coincided with increased SAA. The mare became reluctant to walk, her neck carriage progressively lowered, and she developed a left head tilt resulting in referral to the University of Pennsylvania, New Bolton Center (NBC) for bursoscopy and surgical debridement of the nuchal bursa.

On presentation to NBC, the mare was bright and alert, with normal vital parameters. Mild non-painful swelling was noted over the poll area, most significantly on the right side. Baseline hematology showed moderate hyperfibrinogenemia (605 mg/dl; RI 150–375 mg/dl) and increased SAA (1,205 μg/ml; RI 0–24 μg/ml). Ultrasonographic evaluation revealed synovial proliferation and marked distension of the nuchal bursa with hyperechoic fluid. The dorsal bursa region on the right side of C1 had evidence of fistula formation between the bursa capsule and subcutis. Cytology performed on bursal fluid revealed increased white blood cell count (11,090/μl; RI <500/μl) and total protein (5.1 g/dl; RI <2.0 g/dl). This sample later tested positive by PCR for *B. burgdorferi*. *B. abortus* serology, measured by card agglutination, yielded negative results.

**Figure 3:**
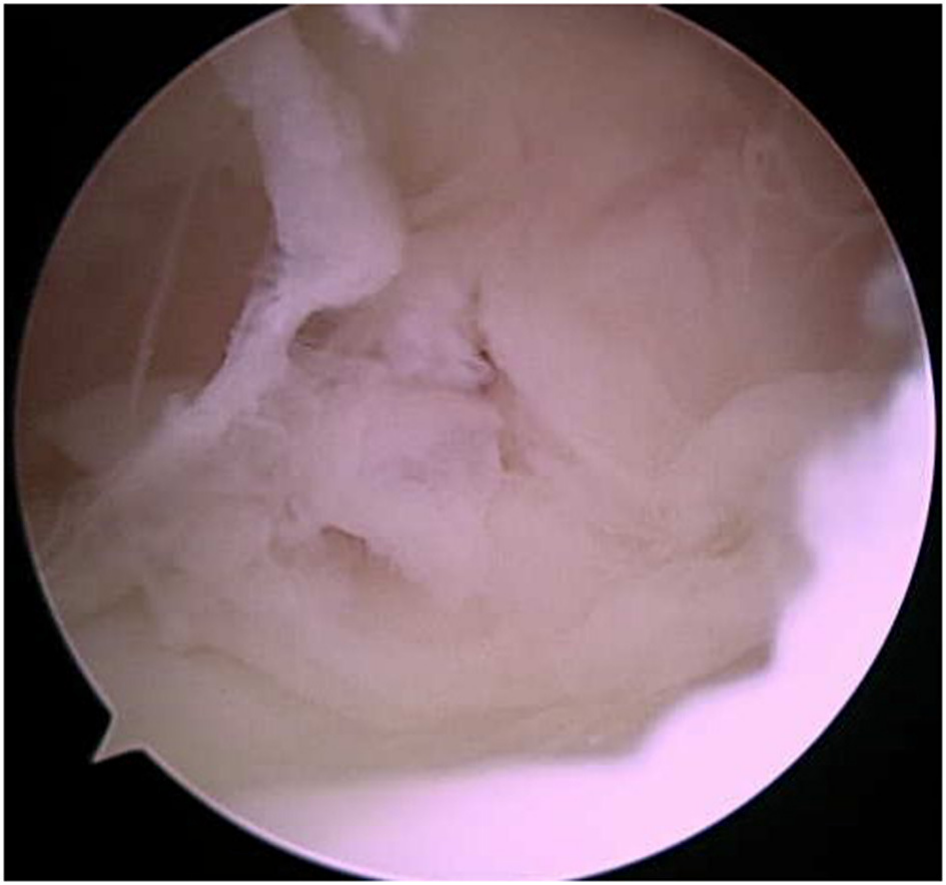
Bursoscopy image showing synovial proliferation within the cranial nuchal bursa.

Endoscopic evaluation of the nuchal bursa was performed under general anesthesia. Moderate synovial proliferation and turbid bursal fluid were noted, which are expected for cases of nuchal bursitis (Figure 3). Copious lavage and synovial debridement using an endoscopic mechanical shaver was performed and the incisions closed routinely. Postoperatively, the mare was maintained on minocycline (4 mg/kg PO 12h) and phenylbutazone (2.2 mg/kg PO q12h). Clinical signs improved initially; however, during the week after discharge, the mare developed fever (102.3°F), facial hyperesthesia and muscle fasciculations, and abnormal vocalization with head movement. Flunixin meglumine (0.5 mg/kg IV) was administered and hematology revealed continued elevation of SAA, moderate lymphopenia (616 cells/μl; RI 1,500–5,500 cells/μl), and mild hyperfibrinogenemia (317 mg/dl; RI 76–230 mg/dl). The bursal fluid was aseptically sampled by the primary care veterinarian and appeared grossly normal. The bursa was then injected with amikacin (Amiglyde, Zoetis, Kalamazoo, MI, 1 g/4 ml) and hyaluronic acid (Hyvisc, Boehringer Ingelheim, 22 mg/2 ml). Due to lack of clinical improvement and continued fever over the following 24 h, the mare returned to NBC for further evaluation. On presentation, she was febrile (101.9°F) with muscle fasciculations on the right side of her face and diffusely across the tongue. She exhibited facial hyperesthesia and resistance to lateral movement and ventroflexion of the neck. A cranial nerve examination showed no loss of function; however, dynamic neurologic gait examination revealed grade 2/5 ataxia of all four limbs on the modified Mayhew scale (15, 16). Hematology showed lymphopenia (540 cells/μl; RI 1,320–5,860 cells/μl), hyperfibrinogenemia (907 mg/dl; RI 150–375 mg/dl), and anemia characterized by low hematocrit (24.5%; RI 30–49%) and low red blood cell count (5,110 cells/μl; RI 5,300–10,500 cells/μl). Cerebrospinal fluid (CSF) was collected via lumbosacral approach and cytology revealed elevated total protein (124 mg/dl; RI <90 mg/dl). Equine protozoal myeloencephalitis snSAG 2, 4/3 ELISA serum:CSF ratio was negative (Equine Diagnostics Solutions, Lexington, KY). Lyme multiplex assay on CSF indicated elevated antibodies directed against the infection markers OspC (1,104 MFI) and OspF (1,362 MFI), while OspA (13,637 MFI) was decreased relative to serum levels, which measured OspA (24,992 MFI; RI <2,000), OspC (213 MFI; RI <1,000 MFI), and OspF (313 MFI; RI <1,250 MFI). *B. burgdorferi* PCR was negative on CSF, but a tentative diagnosis of neuroborreliosis was made based on clinical signs, abnormal CSF cytology and antibody test results, and treatment with ceftiofur sodium (Naxcel, Zoetis, Parsippany-Troy Hills, NJ, 4 mg/kg IV q12h) was initiated. Fever resolved within 24 h and the mare remained hospitalized for 1 week, during which time facial hyperesthesia decreased and neck stiffness improved. The mare was discharged with phenylbutazone (1.1 mg/kg PO q12h), gabapentin (Neurontin, Pfizer, Gladstone, NJ, 17 mg/kg PO q12h), and vitamin E (Elevate W.S., Kentucky Performance Products, Versailles, KY, 10 IU/kg PO q24h), in addition to continued ceftiofur (switched to generic brand after 2 weeks; Ceftiflex, Med-Pharmex Inc., Pomona, CA, 4 mg/kg IV q12h). Over the following 80 days, IV ceftiofur was continued twice daily.

Approximately 8 weeks after discharge, the mare returned for examination. Fibrinogen remained mildly elevated (533 mg/dl; RI 150–375 mg/dl). Palpation of the poll revealed a firm, asymmetrical swelling on the right lateral aspect with surrounding hair loss/dermatitis. An ultrasound of the nuchal bursa was performed, which revealed a moderate amount of echogenic fluid with thick surrounding capsular tissue. Approximately 25 ml of thick, yellow/orange fluid was aseptically obtained from the cranial nuchal bursa under ultrasound guidance. Cytology performed on this fluid revealed increased white blood cell count (75,840 cells/μl; RI <500 cells/μl) and increased total protein (6.1 g/dl; RI <2.0 g/dl); leukocyte differential consisted of 97% poorly preserved and degenerative neutrophils, 1% small lymphocytes, and 2% macrophages amid streaming nuclear debris. The fluid was PCR positive for *B. burgdorferi*, contained antibodies to OspA (24,356 MFI), and no aerobic or anaerobic organisms were obtained in culture. The bursa was injected with 40 mg methylprednisolone acetate (Depo-Medrol; Zoetis) and ceftiofur (1g). Two weeks later, another cranial nuchal bursa ultrasound revealed a subcutaneous pocket of anechoic fluid containing fibrin, with a tract to the cranial nuchal bursa. Aseptic fluid aspiration from the subcutaneous fluid pocket was unsuccessful, and the cranial nuchal bursa was injected with 40 mg methylprednisolone acetate (Depo-Medrol; Zoetis), 2 ml hyaluronate sodium (Hyvisc; Boehringer Ingelheim), and ceftiofur (1 g).

A third and final bursa injection of ceftiofur (1 g) was performed 1 week after IV ceftiofur was discontinued. At that time, the mare had become febrile (101.7°F) and oral minocycline was restarted (4 mg/kg PO q2h). One week later, a draining tract formed and opened above the nuchal bursa; the fluid was PCR positive for *B. burgdorferi*. After the tract drained, fibrinogen levels returned to normal. Minocycline was discontinued 3 months later. A Lyme multiplex assay performed on serum 1 month after discontinuation of minocycline revealed declining OspA antibody values (8,232 MFI; RI <2,000), which continued to decline when tested again after another 3 months (6,255 MFI; RI <2,000). The mare returned to normal work approximately 6 months after initial presentation, with no recrudescence of clinical signs over the following year to date.

## Discussion

Nuchal bursitis is an important differential to consider in the horse when pain and stiffness of the head and neck is observed. Ultrasound imaging is a common modality used in the diagnosis of nuchal bursitis (17), and infectious causes should be considered and investigated.

We have described the involvement of *B. burgdorferi* in a case of equine nuchal bursitis, further supporting the significance of previous findings of *B. burgdorferi* in bursal fluid from horses diagnosed with cranial nuchal bursitis (3). In this case, in addition to identifying *B. burgdorferi* DNA in serial samples of bursal fluid and its associated draining tract, the serologic results provide additional support for this atypical infection. Specifically, the substantially elevated OspA antibody values found in the serum and the bursal fluid suggest the presence of *B. burgdorferi* within the bursa. The reduction in serum OspA antibody values after treatment suggests that clearance of *B. burgdorferi* organism was associated with the resolution of clinical signs.

Antibodies directed against OspA, in the absence of a history of vaccination with this antigen, are typically considered an early infection marker (9, 10); however, this does not encompass the clinical variations observed for these antibody values in practice. The OspA protein, which is expressed to allow adherence and colonization of *B. burgdorferi* in the tick midgut, is downregulated to allow for infection of the mammalian host (18). However, some antigen remains on the surface in the early stage of infection (19), resulting in the modest OspA antibody response observed in the early stage of infection (20). In a field setting, therefore, low positive OspA antibodies might persist as a result of regular exposure to *B. burgdorferi–*infected ticks. However, high OspA antibody values would not be expected in the mammalian host since OspA is not typically produced by *B. burgdorferi* in the course of infection (19, 21). In addition to OspA expression in the tick gut, however, it is known that *B. burgdorferi* readily expresses this protein at other times, in particular, when grown *in vitro* (21). The high positive OspA antibodies detected in the case reported here would have resulted from significant immune stimulation, suggesting that *B. burgdorferi* may have altered its metabolic profile to upregulate OspA expression *in vivo* in this equine patient. Indeed, this alteration in protein expression has been described in a mouse model, where host-adapted *B. burgdorferi* residing in an inflammatory *in vivo* environment upregulated OspA expression (22).

Only three clinical syndromes associated with equine Lyme disease caused by *B. burgdorferi* infection in the horse have been well described in the literature. Neuroborreliosis, the most well-described clinical syndrome, has been associated with the development of neurologic deficits, hyperesthesia, behavioral changes, and neck and back stiffness or pain (23–29). Uveitis can manifest as ocular inflammation secondary to *B. burgdorferi* infection in the eye (11, 30), and a third clinical syndrome associated with equine Lyme disease is cutaneous pseudolymphoma, characterized by skin masses at the site of the tick bite (31, 32). In addition, experimental infection studies revealed that *B. burgdorferi* has a tropism for synovial membranes, and a few case reports associate *B. burgdorferi* infection with synovitis and lameness in the horse (24, 30, 33, 34).

Experimental infection studies in horses, although limited in breadth, do not reveal a high occurrence of clinical disease associated with *B. burgdorferi* infection (32, 35–37). This suggests that horses that develop clinical disease may have some additional underlying condition, such as a co-infection or immunosuppression. In humans, co-infections have been suggested to be associated with Lyme disease; however, this conjecture is not supported by the literature (38, 39). Immunosuppression, specifically common variable immune deficiency, has been associated with cases of neuroborreliosis in horses (25, 29), and immunosuppressed dogs are more susceptible to *B. burgdorferi* infection (40). In the case reported here, the persistent lymphopenia suggests immunosuppression, but it is possible that this hematologic change is related to an immune suppressive mechanism of *B. burgdorferi* (41). It is also possible in the case reported here that the immunomodulatory effects of the previously prescribed medications, such as altrenogest (42, 43) and quercetin (a component of Platinum Skin and Allergy) (44, 45), had an impact on susceptibility to infection; in particular, there is some evidence that flavonoids, such as quercetin, can induce a regulatory immune response, which has the potential to allow for the persistence of pathogen (46). Finally, the horse in this case report had been diagnosed with PPID, a common endocrine disease of older horses associated with immune suppression (47).

Well-defined equine treatment regimens for *B. burgdorferi* infection have not yet been established, and for this reason treatment protocols are currently based on both *in vitro* antibiotic susceptibility data and human protocols (5, 48). Successful treatment of human patients with the neurologic form of Lyme disease has been achieved with parenteral cephalosporin administration (49). Treatment with ceftiofur sodium via both intravenous and intra-bursal routes was implemented in this case due to the bacteriocidal nature of this antibiotic and documented *in vitro* susceptibility of *B. burgdorferi* (50). The prolonged treatment course in this case with intravenous ceftiofur sodium was based on the patient's duration to resolution of clinical signs.

It is important to note that no bacterial organisms were isolated from the bursal fluid samples in this case, and while *B. burgdorferi* is notoriously difficult to culture from the mammalian host (51), *B. burgdorferi* DNA was identified within the bursal fluid. Nucleic acid can persist long after an organism is no longer viable, but the continued presence of *B. burgdorferi* DNA in serial bursal fluid samples supports the existence of this organism at this site of infection. It is unlikely that identification of *B. burgdorferi* DNA was incidental in this case, as it is not common to find *B. burgdorferi* in joint fluid. Internal laboratory data from the Animal Health Diagnostic Center (AHDC) at Cornell University revealed that *B. burgdorferi* DNA was not detected in over 93% of all joint fluid and bursa samples submitted for *B. burgdorferi* PCR testing from 2007 to 2020, while it is estimated that greater than 45% of horses located in the Northeast region of the USA have serologic evidence of infection (52). The reported complaints in horses with *B. burgdorferi* PCR-positive joint fluid and bursa samples submitted to the AHDC included neck pain/stiffness, poll swelling, chronic draining tract at the poll, and acute onset of cranial nuchal bursitis, all of which are reported clinical signs of equine cranial nuchal bursitis, further supporting that *B. burgdorferi* infection can be associated with the pathogenesis of cranial nuchal bursitis in the horse.

In conclusion, Lyme disease is an insidious infection caused by *B. burgdorferi* that is notoriously difficult to diagnose and treat. Typically, antibodies directed against OspC and/or OspF are identified after infection with *B. burgdorferi* in horses, but this case suggests that substantially elevated OspA antibodies alone may be an indication of infection. In cases of suspected clinical Lyme disease, the lack of antibodies against the typical infection markers OspC and OspF may not rule out infection, and in the absence of a history of vaccination against Lyme disease, substantially elevated OspA antibodies may support a diagnosis of Lyme disease and warrant treatment. Finally, infection with *B. burgdorferi* should be considered as a differential in cases of equine septic nuchal bursitis.

## Data Availability Statement

The original contributions presented in the study are included in the article/supplementary material, further inquiries can be directed to the corresponding author/s.

## Author Contributions

CG and TP-W produced the initial manuscript draft. All authors reviewed, contributed, and approved the final version for publication. AJ oversaw patient care in hospital, JM oversaw patient care on farm, and DL performed the surgery.

## Conflict of Interest

The authors declare that the research was conducted in the absence of any commercial or financial relationships that could be construed as a potential conflict of interest.

## Publisher's Note

All claims expressed in this article are solely those of the authors and do not necessarily represent those of their affiliated organizations, or those of the publisher, the editors and the reviewers. Any product that may be evaluated in this article, or claim that may be made by its manufacturer, is not guaranteed or endorsed by the publisher.
